# Implication of an Aldehyde Dehydrogenase Gene and a Phosphinothricin *N*-Acetyltransferase Gene in the Diversity of *Pseudomonas cichorii* Virulence

**DOI:** 10.3390/genes3010062

**Published:** 2011-12-27

**Authors:** Masayuki Tanaka, Ullah Md Wali, Hitoshi Nakayashiki, Tatsuya Fukuda, Hiroyuki Mizumoto, Kouhei Ohnishi, Akinori Kiba, Yasufumi Hikichi

**Affiliations:** 1 Laboratory of Plant Pathology and Biotechnology, Kochi University, Nankoku, Kochi 783-8502, Japan; E-Mails: tanakamasayukikani@yahoo.co.jp (M.T.); wullahsau@yahoo.com (U.M.W.); jm-mizumoto@kochi-u.ac.jp (H.M.); akiba@kochi-u.ac.jp (A.K.); 2 Laboratory of Plant Pathology, Kobe University, Kobe, Hyogo 657-8501, Japan; E-Mail: hnakaya@kobe-u.ac.jp; 3 Faculty of Agriculture, Kochi University, Nankoku 783-8502, Japan; E-Mail: tfukuda@kochi-u.ac.jp; 4 Research Institute of Molecular Genetics, Kochi University, Nankoku, Kochi 783-8502, Japan; E-Mail: kouheio@kochi-u.ac.jp

**Keywords:** *Pseudomonas cichorii*, *aldH*, *pat*, *hrp*, virulence, diversity

## Abstract

*Pseudomonas cichorii* harbors the *hrp* genes. *hrp*-mutants lose their virulence on eggplant but not on lettuce. A phosphinothricin *N*-acetyltransferase gene (*pat*) is located between *hrpL* and an aldehyde dehydrogenase gene (*aldH*) in the genome of *P. cichorii*. Comparison of nucleotide sequences and composition of the genes among pseudomonads suggests a common ancestor of *hrp* and *pat* between *P. cichorii* strains and *P. viridiflava* strains harboring the single *hrp* pathogenicity island. In contrast, phylogenetic diversification of *aldH* corresponded to species diversification amongst pseudomonads. In this study, the involvement of *aldH* and *pat* in *P. cichorii* virulence was analyzed. An *aldH*-deleted mutant (ΔaldH) and a *pat*-deleted mutant (Δpat) lost their virulence on eggplant but not on lettuce. *P. cichorii* expressed both genes in eggplant leaves, independent of HrpL, the transcriptional activator for the *hrp*. Inoculation into *Asteraceae* species susceptible to *P. cichorii* showed that the involvement of *hrp*, *pat* and *aldH* in *P. cichorii* virulence is independent of each other and has no relationship with the phylogeny of *Asteraceae* species based on the nucleotide sequences of *ndhF* and *rbcL*. It is thus thought that not only the *hrp* genes but also *pat* and *aldH* are implicated in the diversity of *P. cichorii* virulence on susceptible host plant species.

## 1. Introduction

*Pseudomonas cichorii* causes bacterial rot in lettuce, which is characterized by shiny, dark brown, firm necrotic spots on leaves underneath the second or the third outermost head-leaves [1,2,3]. *P. cichorii* reportedly causes midrib rot of greenhouse-grown butterhead lettuce [4].

In culture medium and on lettuce leaves, *P. cichorii* does not produce pectate lyase, the most important extracellular plant cell wall-degrading enzyme. The development of disease symptoms is closely associated with programmed cell death (PCD) following heterochromatin aggregation and laddering of genomic DNA in the *P. cichorii*-infected lettuce cells [5]. *P. cichorii* also causes necrotic spots on eggplant distinct from the disease symptoms on lettuce [6,7]. Kiba *et al*. [7] also showed that development of necrotic spot symptoms following PCD in leaves of eggplant infiltrated with *P. cichorii* was associated with *de novo* protein synthesis, intracellular reactive oxygen species and caspase III-like proteases.

In several Gram-negative phytopathogenic bacteria, the *hrp* genes (*hrp*) are essential determinants for disease development in compatible hosts and for elicitation of the hypersensitive response (HR) on resistant plants [8]. The *hrp* cluster encodes proteins of the type III secretion system (T3SS), which transports virulent proteins directly into the host cells. These proteins subsequently cause leakage of plant nutrients into the extracellular spaces of infected tissues and suppress host defenses. Nine of the *hrp* have been renamed *hrc* (HR and conserved) to indicate that they encode conserved components that are also present in T3SS of the animal pathogens *Yersinia*, *Shigella* and *Salmonella* [9].

The *hrp* cluster reportedly exists in the genomic DNA of *P. cichorii* [6,10]. *hrp*-deficient mutants of SPC9018 grow slowly, and the appearance of disease symptoms on infected lettuce leaves is delayed compared with the wild type strain, suggesting that the putative T3SS-dependent effector proteins may hinder or delay the plant defense response, giving the bacteria time to multiply before inducing PCD in lettuce leaves [6]. On the other hand, *hrp*-deficient mutants lose both their ability to vigorously grow in eggplant leaves and their virulence on eggplant. It is thus thought that the *hrp* cluster may be implicated in the diversity of *P. cichorii* virulence.

Nucleotide sequences and gene composition of the *hrp* cluster in *P. cichorii* are homologous to those in the single pathogenicity island (S-PAI) of *P. viridiflava* [6,10]. Furthermore, Hojo *et al*. [6] demonstrated functional conservation of *hrpF* operons between *P. cichorii* strain SPC9018 (SPC9018) and *P. viridiflava* strain Pv9504 (Pv9504) harboring the S-PAI. These lines of evidence suggest a common ancestor for the *hrp* cluster between *P. cichorii* strains and the S-PAI of *P. viridiflava*.

**Figure S1:**
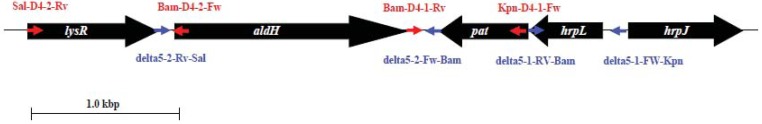
Assembly of open reading frames of the DNA region in the flanking region of *hrp* of *P. cichorii* strain SPC9018 genomic DNA including *aldH* and *pat* and position of primers used for constructions of plasmids for the *aldH-*deleted mutant (red-colored) and the *pat*-deleted mutant (blue-colored).

A phosphinothricin *N*-acetyltransferase gene (*pat*) is located between *hrpL* and an aldehyde dehydrogenase gene (*aldH*) in genomic DNA of *P. cichorii* (Figure S1) and the BS and AS groups of *P. viridiflava* strains harboring S-PAI but not the AT group of *P. viridiflava* strains harboring the tripartite pathogenicity island (T-PAI) and *P. syringae* strains [6,10,11]. It is thought that there is a common ancestor of *pat* between *P. cichorii* strains and the BS and the AS group strains harboring the S-PAI in *P. viridiflava* [10].

Wang *et al.* [12] reported that *aldH* is involved in the virulence of *Agrobacterium tumefaciens* in response to starvation orhost signals. Moreover, the ToxR-regulated *aldH* of epidemic and pandemic strains of *V. cholerae* is located at the left end of a chromosomal PAI, adjacent to a putative transposase gene which is present in epidemic and pandemic strains but absent from nonpathogenic strains [13]. The PAGI-1 region is reportedly inserted within the 3' region of *aldH* in genomic DNA of virulent *P. aeruginosa* strain X24509 [14]. Deduced amino acid sequences of the protein encoded by *pat* showed a similarity to those of phosphinothricin acetyltransferase, which is encoded by *bar* and specifically acetylates L-phosphinothricin that shows toxicity against microorganisms and plants, leading to resistance of the bacteria to L-phosphinothricin [15,16,17]. Though the available evidence suggests *aldH* and *pat* may be involved in environmental responses, we have no information on the involvement of *aldH* and *pat* in *P. cichorii* virulence. In this study, we thus analyzed the involvement of *aldH* and *pat* in the diversity of *P. cichorii* virulence on susceptible host plant species.

## 2. Results and Discussion

### 2.1. *In Vitro* Growth of the *aldH*-Deficient Mutant and the *pat*-Deficient Mutant of P. cichorii

**Figure 1:**
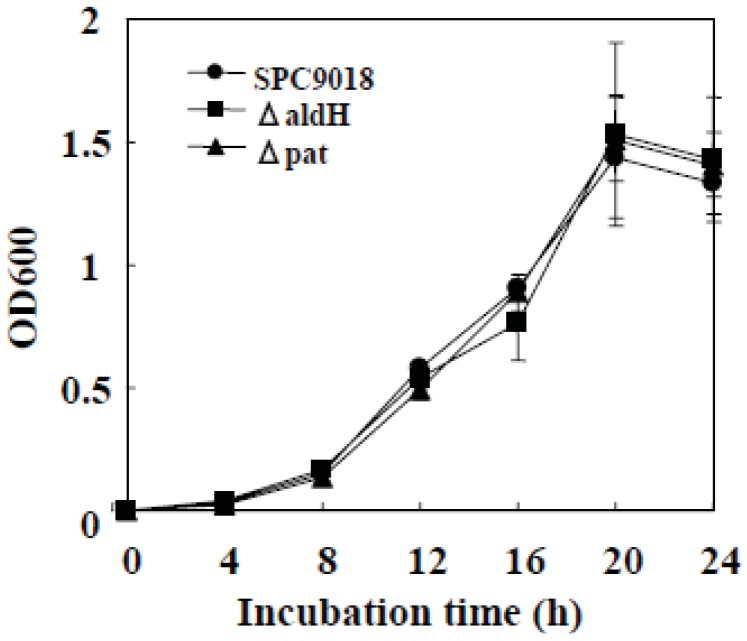
Growth of *P. cichorii* strains incubated in PY-medium.

To compare the *in vitro* growth ability of the *aldH*-deficient mutant (ΔaldH) and the *pat*-deficient mutant (Δpat) from SPC9018 with that of SPC9018, *P. cichorii* strains were incubated in PY-medium and the optical density at 600 nm (OD600) of the bacterial suspensions was measured. The growth rate of both ΔaldH and Δpat was equal to that of SPC9018, showing that deletion of *aldH* and *pat* does not affect *in vitro* growth of the bacteria (Figure 1).

### 2.2. Involvement of *aldH* and *pat* in Virulence of P. cichorii on Eggplant but not on Lettuce

**Figure 2:**
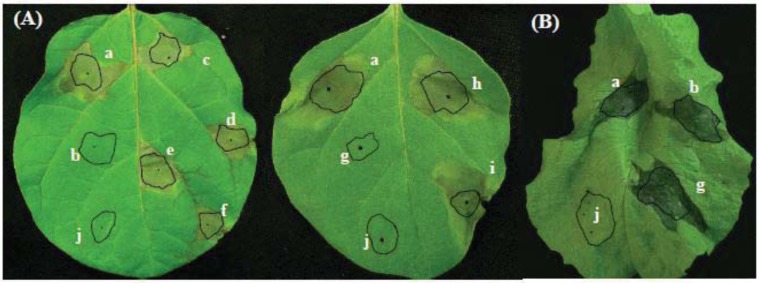
(**A**) Necrotic lesions on eggplant leaves three days post-inoculation, and (**B**) rot on lettuce leavesone day post-inoculation with *P. cichorii.* Strains (a) SPC9018; (b) ΔaldH; (c) ΔaldH(Pc-aldH); (d) ΔaldH(Pv-aldH); (e) ΔaldH(Pst-aldH); (f) ΔaldH(Pa-aldH); (g) Δpat;(h) Δpat(Pc-pat); (i) Δpat(Pv-pat): were inoculated at a bacterial density of 1.0 × 10^8^ cfu/mL in distilled water in a 20 μL volume (circled); Leaves were inoculated with distilled water (j) as a negative control. Plants were grown at 25 °C (10,000 L × 16 h/day).

When inoculated with SPC9018, symptoms on leaves of eggplant and lettuce developed within one day post-inoculation and then progressed beyond the inoculated area within three days post-inoculation (Figure 2). The ΔaldH and the Δpat mutants lost their virulence on eggplants (Figure 2A). However, both mutants exhibited virulence on lettuce, similar to SPC9018 (Figure 2B). To confirm the involvement of *aldH* and *pat* in *P. cichorii* virulence against eggplant, the ΔaldH and Δpat mutants were complemented with plasmids pPc-aldH and pPc-pat carrying *aldH* and *pat*, respectively, from the SPC9018 genome. The transformants, ΔaldH(Pc-aldH) and Δpat(Pc-pat), exhibited virulence against eggplant similar to SPC9018 (Figure 2A). These results indicate involvement of *aldH* and *pat* in SPC9018 virulence on eggplant but not on lettuce.

### 2.3. Involvement of *pat*, but not *aldH*, in Bacterial Growth in Planta

**Figure 3:**
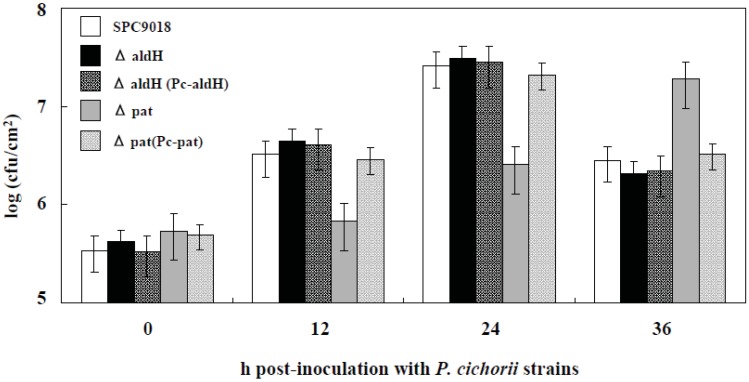
Population dynamics of *P. cichorii* strains in infiltrated eggplant leaves. Values represent the mean ± standard deviation (SD) of five separate experiments. Populations of ΔaldH, ΔaldH(Pc-aldH) and Δpat(Pc-pat) were not significantly different from the population of *P. cichorii* strain SPC9018 (P < 0.05) by the Student’s t-test.

The ΔaldH mutant grew vigorously in the inoculated area of eggplant leaves, and reached a maximum population size of 2.6 × 10^7^ cfu/cm^2^ at 24 h post-inoculation, similar to SPC9018 and the complemented strain ΔaldH(Pc-aldH) (Figure 3). In contrast, the Δpat mutant grew slower compared to SPC9018 and reached a maximum population size of 1.9 × 10^7^ cfu/cm^2^ at 36 h post-inoculation. The complemented strain Δpat(Pc-pat) grew similarly to SPC9018.

The deduced amino acid sequences based on the nucleotide sequences of *aldH* and *pat* showed that the amino acid sequences characteristic of type III effectors [18] were not observed in the *N*-terminal of the proteins encoded in *aldH* and *pat*. The PSORT prediction of deduced amino acid sequences of the proteins encoded in *aldH* and *pat* showed localization in the bacterial cytoplasm and the bacterial inner membrane, respectively. Though the PSORT prediction is limited to identify secreted proteins through the Sec pathway, it is thought that SPC9018 may not extracellularly secrete both proteins.

Results in this study indicate involvement of *pat* but not *aldH* in bacterial growth in eggplant leaves. Hojo *et al*. [6] indicate that vigorous growth of SPC9018 in eggplant leaves immediately after invasion may be involved in its virulence. This evidence leads us to consider that the protein encoded in *pat* might be implicated in the rapid growth of *P. cichorii* immediately after invasion into eggplant leaves, and may be associated with stressors or signals present in eggplant but not in lettuce. Wang *et al.* [12] reported that mutation of *aldH* in *Agrobacterium tumefaciens* resulted in early expression of the quorum sensing signal degrading enzyme, AttM. Furthermore, aldehyde dehydrogenase is reportedly a member of virulence regulons in *V. cholerae* [13]. It is thus thought that either the signal transduction through the protein encoded in *aldH* or the protein itself might be involved in functional regulation of virulence factors such as quorum sensing, which are involved in induction of PCD in eggplant but not in lettuce. Further analysis of *aldH* and *pat* will be required for elucidation of the virulence mechanism of SPC9018, as well as genetic and functional information regarding type III effectors.

### 2.4. Expression of *aldH* and pat is not Regulated by HrpL

**Figure 4:**
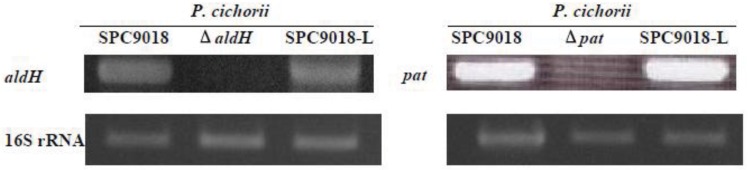
Reverse transcription-polymerase chain reaction analysis of *aldH* and *pat* of *P. cichorii* strains infecting eggplant leaves. Total RNA was isolated from eggplant leaves at 8 h post-infiltration with *P. cichorii* strains.

HrpL, a member of the ECF family of alternative sigma factors [19], activates the *hrp*, *hrc* and type III effector genes of pseudomonads [6,10]. To analyze the dependency of *aldH* and *pat* expression on HrpL in *P. cichorii*, the expression of *aldH* and *pat* was examined by RT-PCR on samples taken at 8 h post-inoculation. In inoculated eggplant leaves, the expression of *aldH* and *pat* in the *hrpL*-deleted mutant, SPC9018-L [6] was similar to the parent strain SPC9018 (Figure 4). When RNA treated with DNase I was used as template in PCR reactions, no product was observed.

HrpL is thought to bind to a consensus bipartite *cis* element (*hrp* box) present in the promoter region of the *hrp*, *hrc* and type III effector genes [19,20,21]. The hrp box (GGAACC-N15-16-CCANNCA) exists in the promoter regions of *hrpA*, *hrpF*, *hrpW*, *avrF*, *avrE* and *hrpJ* in the genomic DNA of *P. cichorii*, and expression of these genes, is dependent on HrpL [6,10]. Absence of the *hrp* box in the promoter region of *aldH* and *pat* and results in this study indicate that SPC9018 expresses *aldH* and *pat* independently of HrpL.

### 2.5. Phylogenetic Diversity and Functional Conservation of *aldH* among Pseudomonads

**Figure 5:**
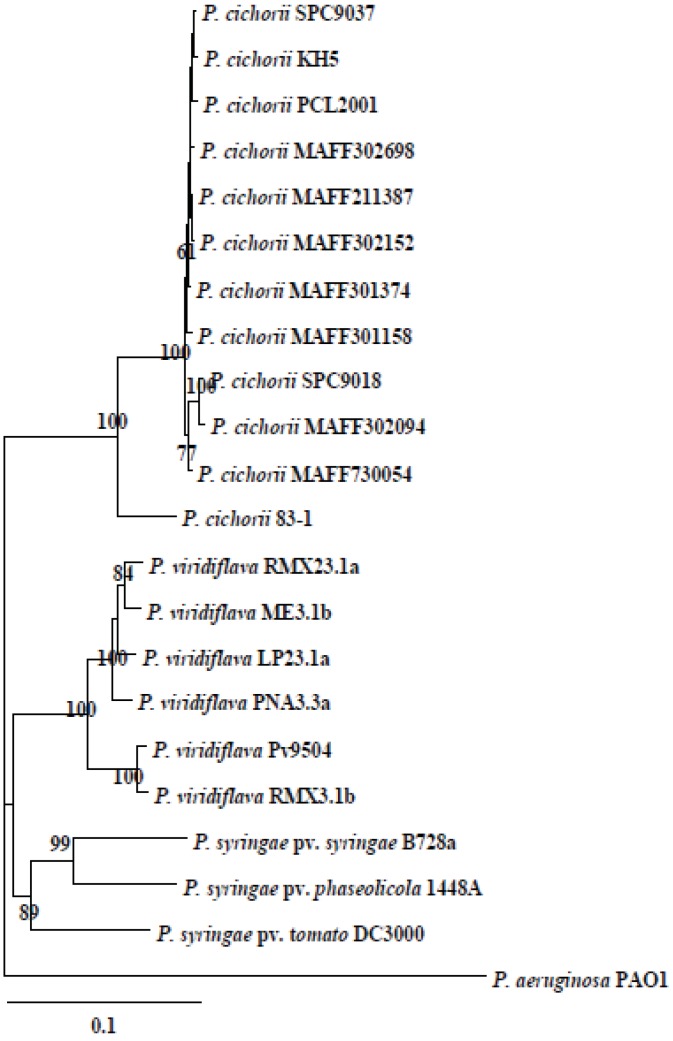
Phylogenetic trees based on the nucleotide sequences of *aldH*. Phylogenetic trees were constructed using ClustalW (DNA Data Bank of Japan [22]) employing the Neighbor-Joining method [23]. The scale bar indicates a genetic distance, which is the expected number of substitutions per position. The numbers at the nodes represent percentage bootstrap values of 1,000 resamplings that exceeded 60%. The nucleotide sequences of *aldH* from *P. aeruginosa* strain PAO1 were used for the phylogenetic tree reconstruction.

*aldH* is also located in the genomic DNA of other pseudomonads such as *P. viridiflava* and *P. syringae* strains [10,11]. To compare the phylogenetic diversity between *aldH*, nucleotide sequences (Table S1) of *aldH* from *P. cichorii*, *P. viridiflava* and *P. syringae* were analyzed. In the phylogenetic tree based on the nucleotide sequence of *aldH*, all these species represent different clusters (Figure 5).

To analyze functional conservation of *aldH* among pseudomonads, the ΔaldH mutant was transformed with *aldH* genes originating from the *P. viridiflava* BS group strain Pv9504, *P. syringae* pv. *tomato* strain DC3000 and *P. aeruginosa* strain PAO1, creating ΔaldH(Pv-aldH), ΔaldH(Pst-aldH) and ΔaldH(Pa-aldH), respectively. All transformants exhibited virulence on eggplant, similar to ΔaldH(Pc-aldH) and SPC9018 (Figure 2A). The virulence of these transformants suggests that *aldH* is conserved amongst pseudomonads and that phylogenetic diversification of *aldH* may correspond to species diversification.

### 2.6. Functional Conservation of *pat*

*pat* is located in the genome of *P. cichorii* strains and the S-PAI of *P. viridiflava* AS and BS group strains [10]. To analyze the functional conservation of *pat* between SPC9018 and Pv9504, the Δpat mutant was transformed with *pat* originating from *P. viridiflava* strain Pv9504. The transformant, Δpat(Pv-pat) exhibited virulence on eggplant similar to SPC9018 (Figure 2A). The virulence of this transformant suggests functional conservation of *pat* between *P. cichorii* and *P. viridiflava* BS and AS group strains harboring the S-PAI, supporting a common ancestry of *pat* between *P. cichorii* strains and the BS and AS group strains of *P. viridiflava*, as well as the *hrp* genes.

The composition of bacterial genomes can change rapidly and dramatically through a variety of processes, including horizontal gene transfer [24] which has been recognized as the universal event throughout bacterial evolution [14,25]. Acquiring horizontally transferred genes is an efficient way to alter the genotype of a bacterium, leading to the creation of a new phenotype or even a new species [26,27]. Although a single gene might have a low horizontal transfer index (HTI) purely by chance, it is unlikely that a large cluster of neighboring genes would all have low HTIs by chance. Such clusters are considered to be a single unit simultaneously inserted into the genome [28]. In particular, it has been suggested that a number of pathogenicity genes were horizontally transferred as large clusters, PAIs [29]. It is thus thought that a pathogenicity island in SPC9018 consists of the *hrp* and *pat*. The pathogenicity island with *aldH* is implicated in virulence of SPC9018 on eggplant but not on lettuce.

### 2.7. Implication of *hrp*, *aldH* and *pat* in SPC9018 Virulence on Asteraceae Plants

**Figure 6:**
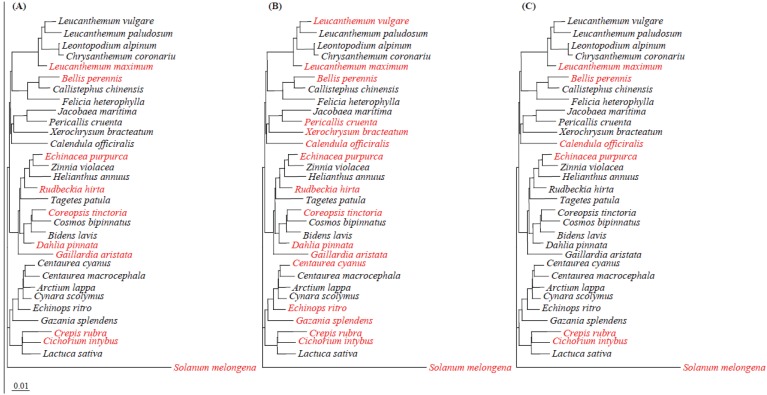
Relationship between the phylogenetic tree of *Asteraceae* plants based on the combined partial nucleotide sequences of *ndhF* and *rbcL* and virulence of SPC9018-hrcC (**A**), ΔaldH (**B**) and Δpat (**C**) on *Asteraceae* plants. The phylogenetic tree was constructed with Clustalwusing the NJ method [22,23]. The scale bar indicates genetic distance, which is the expected number of substitutions per position. *P. cichorii* strains showed virulence and non-virulence on plants with black-colored and red-colored letters, respectively.The nucleotide sequences of the combined partial nucleotide sequences of *ndhF* and *rbcL* from eggplant (*Solanum melongena*) were used as the outgroup for phylogenetic tree reconstructions.

Virulence analysis showed that deletion of *aldH* and *pat* resulted in loss of SPC9018 virulence on eggplants but not on lettuce. When 31 species of *Asteraceae* plants (Table S2) including lettuce (*Lactuca sativa*) were inoculated with SPC9018, we could observe necrotic regions on leaves of all plant species at three days post-inoculation, indicating that SPC9018 is virulent on all *Asteraceae* plants. To analyze the involvement of *hrp*, *aldH* and *pat* in SPC9018 virulence on the *Asteraceae* species, we inoculated the *Asteraceae* plants with a *hrcC*-mutant, SPC9018-hrcC, and with the ΔaldH and Δpat mutants. The mutants, SPC9018-hrcC, ΔaldH and Δpat lost their virulence on 9, 16 and 6 species, respectively (Figure 6 and Table S2). Between them, all mutants lost their virulence on 5 species.

To determine the relationship between the involvement of *hrp*, *aldH* and *pat* in *P. cichorii* virulence on these plants and phylogeny among the *Asteraceae* species, phylogenetic trees were constructed based on the combined nucleotide sequences of *ndhF* and *rbcL* (Figure 6 and Table S2). The NJ phylogenetic tree placed 31 species in two major clusters (Figure 6). Involvement of *hrp*, *aldH* and *pat* in SPC9018 virulence on the respective species had no relationship with phylogeny of the *Asteraceae* species. It is thus thought that the implication of not only *hrp* but also *aldH* and *pat* in *P. cichorii* virulence might have arisen after species diversification of *Asteraceae* plants and be responsible for the virulence of *P. cichorii* towards respective species.

Araki *et al.* [10] and Hojo *et al*. [6] demonstrated that *P. cichorii* strains acquired the *hrp* genes through horizontal transfer from a common ancestor with the S-PAI of *P. viridiflava* and implicated the *hrp* in its virulence. Symptoms on *P. cichorii*-infected lettuce leaves are characterized by shiny, dark brown, firm necrotic spots [2,3]. *P. cichorii* also causes necrotic spots on eggplant distinct from the disease symptoms on lettuce leaves [6,7]. The development of disease symptoms on both lettuce leaves and eggplant leaves is closely associated with PCD in *P. cichorii*-infected tissues [5,7]. Virulence of *P. cichorii* on eggplant is *hrp*-dependent. On the other hand, the *hrp* are not directly implicated in induction of PCD in infected lettuce leaves and the disease development on lettuce leaves caused by the *hrp*-deficient mutants is delayed compared with that caused by the parent strain, since the *hrp* genes play a role in *P. cichorii* growth at the early infection stages in lettuce leaves [6]. From these evidences, Hojo *et al*. [6] demonstrate that the effect of the *hrp* on *P. cichorii* virulence differs between lettuce and eggplant. Results in this study suggested conservation of *aldH* amongst pseudomonads and supported a common ancestor of *pat* between *P. cichorii* and *P. viridiflava* strains harboring the S-PAI. Furthermore, results in this study implicate not only the *hrp* genes but also *aldH* and *pat* in the diversity of its virulence towards susceptible host plants. Therefore, it is hypothesized that *P. cichorii* might maintain the *hrp*, *aldH* and *pat* to establish its virulence on respective host plants.

## 3. Experimental Section

### 3.1. Bacterial Strains, Plasmids and Culture Conditions

The bacterial strains and plasmids used in this study are listed in Table S3. *P. cichorii*, *P. viridiflava*, *P. syringae* pv. *tomato* and *P. aeruginosa* strains were routinely grown in PY-medium (5% polypeptone, 2% yeast extract) at 30 °C. *Escherichia coli* strains were grown in LM medium [30] at 37 °C. The optimal density at 600 nm (OD600) of the bacterial suspensions was measured with Ultrospec 1100pro (GE Healthcare, Tokyo, Japan). Ampicillin (50 μg/mL, nakalai tesque, Kyoto, Japan), chloramphenicol (50 μg/mL, nakalai tesque), kanamycin (50 μg/mL, nakalai tesque) and tetracycline (30 μg/mL, nakalai tesque) were used in selective media. Populations of SPC9018 and mutants *in planta* were assayed in three independent experiments using PCSM plates [31] and PCSM plates containing appropriate antibiotics.

### 3.2. DNA Manipulations

Isolation of genomic DNA, plasmid DNA manipulations, and PCR analysis were performed using standard techniques [6,32]. *P. cichorii* was transformed by electroporation as described by Hojo *et al*. [6]. Double-stranded DNA sequencing templates were prepared with GenElute™ Plasmid Miniprep Kits (SIGMA Chemical, St. Louis, MO, USA). Sequences were determined using an ABI Prism 3100-Avant Genetics Analyzer (Applied Biosystems, Tokyo, Japan). DNA sequence data were analyzed using the DNASIS-Mac software (Hitachi Software Engineering, Yokohama, Japan). Enzymes including restriction endonucleases (Takara, Ohtsu, Japan) and Ex-taq polymerase (Takara) were used according to the manufacturer’s instructions. The primers used in this study are listed in Table S4.

### 3.3. Creation of *aldH-* and *pat*-Deleted Mutants

Plasmids designed to create deletion mutants using the kanamycin-resistant gene originating from pUCK191 [33] were based on pHSG398 (Takara) ligated *sacB* originating from pUCD800 [34]. The construction of the clones is described in detail in Supplementary Materials. The resulting plasmids, pD4-1-2KmSacB and pD5-1-2KmSacB, were electroporated into SPC9018 cells and the resultant kanamycin- and sucrose-resistant, and chloramphenicol-sensitive recombinants, an *aldH*-deficient mutant (ΔaldH) and a *pat*-deficient mutant (Δpat), were selected, respectively.

### 3.4. Creation of the *hrcC*-Mutant

For creation of the *hrcC*-mutant, a plasmid, pHSG398sacBhrcCKm, was created. The construction of the clones is described in detail in Supplementary Materials. The plasmid pHSG398sacBhrcCKm was electroporated into SPC9018 cells and a resultant kanamycin and sucrose-resistant recombinant, SPC9018-hrcC, was selected.

### 3.5. Complementation of ΔaldH with *aldH* Originating from Pseudomonads

For complementation of ΔaldH with *aldH* originating from pseudomonads, the plasmids, pPc-aldH, pPst-aldH and pPa-aldH, were created. A detailed cloning procedure is described in Supplementary Materials. The plasmids pPc-aldH, pPst-aldH and pPa-aldH containing *aldH*, originating from SPC9018, *P. syringae* pv. *tomato* strain DC3000 and *P. aeruginosa* strain PAO1, were transformed into ΔaldH competent cells and chloramphenicol-resistant transformants, ΔaldH(Pc-aldH), ΔaldH(Pst-aldH) and ΔaldH(Pa-aldH) were created, respectively. The plasmid pPv-aldH was also transformed into ΔaldH competent cells to create a tetracycline-resistant ΔaldH(Pv-aldH) transformant.

### 3.6. Complementation of Δpat with *pat* Originating from SPC9018 and Pv9504

For complementation of Δpat with *pat* originating from SPC9018 and Pv9504, the plasmids, pPc-pat and pPv-pat, were created, respectively. A detailed cloning procedure is described in Supplementary Materials. The plasmids pPc-pat and pPv-pat including *pat*, originating from SPC9018 and Pv9504, were transformed into Δpat competent cells and a chloramphenicol-resistant Δpat(Pc-pat) transformant and a chloramphenicol-resistant Δpat(Pv-pat) transformant, were created, respectively.

### 3.7. Expression Analysis of *aldH* and *pat* in P. cichorii Strains by RT-PCR

To analyze the expression of *aldH* and *pat* by reverse transcription-PCR (RT-PCR), total RNA was isolated from five of each set of the bacteria-infiltrated area in eggplant leaves (0.6 g) 8 h after infiltration with with 50 μL of the bacterial suspension (1.0 × 10^8^ cfu/mL) of *P. cichorii* strains, and DNase I (Applied Biosystems, Tokyo, Japan) treatment was used to remove the genomic DNA from the RNA preparation [35]. The cDNA of *aldH* and *pat* was synthesized from total RNA (6 μg) using reverse transcription RAV-2 (Takara) utilizing primers SEMI-Back and ORF5-RT-Rv, respectively. The PCR was carried out with the primers: SEMI-Front and SEMI-Back to amplify a 500 bp amplicon specific to *aldH*; and ORF5-RT-Fw and ORF5-RT-Rv to amplify a 450 bp DNA fragment specific to *pat*. The cDNA of 16S rRNA was synthesized utilizing a primer, 16S-rRNA-Rv. The PCR was carried out with primers 16S-rRNA-Fw and 16S-rRNA-Rv to amplify a 448 bp amplicon specific to 16S rRNA.

### 3.8. Sequencing of *aldH* from P. cichorii Strains

A 1.7 kb DNA fragment containing *aldH* was amplified from the genomic DNA of *P. cichorii* strains (Table S2) and sequenced with aldH-Fw1 and aldH-Rv as primers.

### 3.9. Sequencing of *ndhF* and *rbcL* from Asteraceae Plants

The partial *ndhF* and *rbcL* fragments were amplified by PCR from the genomic DNA of 31 species of *Asteraceae* plants (Table S2) using the primers ndhF-11FW and ndhF-22RV for *ndhF*; and 1-1 and NN3-2 for *rbcL*. The nucleotide sequences of partial *ndhF* and *rbcL* DNA fragments were determined directly from the PCR fragments using ndhF-11FW, ndhF-22RV, ndhF-11RV, ndhF-22FW, 1-2FW, 1-2RV, 1-1 and NN3-2.

### 3.10. Data Analysis

The nucleotide sequences of *aldH* (1,584 bp), and combined *ndhF* (1,996–2,023 bp) and *rbcL* (1244–1247 bp) were aligned and phylogenetic trees were constructed using ClustalW by the neighbor-joining (NJ) method [22,23]. Genetic distances were computed with Kimura’s two-parameter model [39]. The NJ phylogenetic tree was drawn by TreeView.

### 3.11. Virulence Assays

Eggplant (*Solanum melongena* L. cv. Senryo No. 2) and the plants listed in Table S2, including lettuce (*Lactuca sativa* L. cv. Success) were grown in pots containing a high-grade potting mix (Tsuchitaro; Sumitomo Forestry Co. Ltd., Tokyo, Japan) at 25 °C. Light (16 h/day) was supplied at 10,000 L × throughout the experimental period. Five-week-old test plants were inoculated by leaf-infiltration using a 1 mL disposable syringe with 1.0 × 10^8^ cfu/mL bacteria in a 20 μL volume. Following inoculation, plants were incubated for 3 days under 100% relative humidity at 25 °C and then grown at 25 °C. For all assays, inoculum concentrations were determined spectrophotometrically and confirmed by dilution plating. Plants were inspected for symptoms daily for five days after inoculation. We replicated virulence assays on tested species at five trials. Within each trial, 12 plants for each strain were treated, yielding 60 plants per strain.

### 3.12. Bacterial Population in Planta

Areas (1 cm^2^) inoculated with *P. cichorii* strains were excised from the eggplant leaves of five plants at 0, 12, 24 and 36 h post-inoculation and ground using a mortar and pestle. Samples (0.1 mL) of the original solution and 10-fold serial dilutions were spread onto three plates of selective agar media containing appropriate antibiotics. Colonies were counted after 2 days of incubation at 30 °C to estimate the population.

### 3.13. Nucleotide Sequence Accession Numbers

The nucleotide sequences determined in this study have been deposited in the DDBJ/GenBank international nucleotide sequence database under the accession numbers shown in Table S1 and Table S5.

## 4. Conclusions

It is thought that not only the *hrp* genes but also *pat* and *aldH* are implicated in the diversity of *P. cichorii* virulence on susceptible host plant species. 

## Supplementary Material

### 1. Construction of Plasmids for *aldH* and *pat*-Deletion Mutants

To construct a plasmid, pD4-1-2KmSacB, for creation of an *aldH*-deleted mutant (ΔaldH) from SPC9018, a 780-bp fragment, designated D4-1, was first amplified by PCR from the genomic DNA of SPC9018 with the primers Kpn-D4-1-Fw and Bam-D4-1-Rv (Figure S1). The *Bam*HI and *Kpn*I-digested D4-1 was ligated into *Bam*HI and *Kpn*I sites of pHSG398 and the resulting plasmid was designated pD4-1. A 1,080-bp fragment, designated D4-2, was PCR-amplified from the genomic DNA of SPC9018 with the primers Bam-D4-2-Fw and Sal-D4-2-Rv. The *Sal*I- and *Bam*HI-digested D4-2 was ligated into the *Sal*I and *Bam*HI sites of pD4-1 and the resulting plasmid was designated pD4-1-2. A 1.4-kb fragment, designated Bam-Km, was PCR amplified from the DNA of pUCK191 with the primers Bam-Km1 and Bam-Km2. The *Bam*HI-digested Bam-Km was ligated into the *Bam*HI site of pD4-1-2 and the resulting plasmid was designated pD4-1-2Km. A 2.6-kb fragment, designated Sal-SacB, was amplified via PCR from pUCD800 DNA with the primers Sal-SacB1 and Sal-SacB2. The *Sal*I-digested Sal-SacB was ligated into the *Sal*I site of pD4-1-2Km and pD4-1-2KmSacB was constructed.

To construct pD5-1-2KmSacB for creation of a *pat*-deleted mutant (Δpat) from SPC9018, a 906-bp fragment, designated D5-1, was amplified by PCR from the genomic DNA of SPC9018 with the primers delta5-1-FW-Kpn and delta5-1-RV-Bam (Figure S1). The *Bam*HI and *Kpn*I-digested D5-1 was ligated into the *Bam*HI and *Kpn*I sites of pHSG398 and the resulting plasmid was designated pD5-1. A 1,949-bp fragment, designated D5-2, was PCR-amplified from the genomic DNA of SPC9018 with the primers delta5-2-Fw-Bam and delta5-2-Rv-Sal. The *Sal*I- and *Bam*HI-digested D5-2 was ligated into the *Sal*I and *Bam*HI sites of pD5-1 and the resulting plasmid was designated pD5-1-2. The *Bam*HI-digested Bam-Km was ligated into the *Bam*HI site of pD5-1-2 and the resulting plasmid was designated pD5-1-2Km. The *Sal*I-digested Sal-SacB fragment was ligated into the *Sal*I site of pD5-1-2Km and pD5-1-2KmSacB was constructed.

### 2. Construction of Plasmids for the *hrcC*-Mutant

The pUCD800 was digested with *Bam*HI and *Pst*I resulting in a 2.6-kb DNA fragment containing *sacB* that was ligated into the *Bam*HI and *Pst*I sites of pHSG398 to create pHSG398sacB. The plasmid, phrpFoperon [6], containing the *hrpF* operon was digested with *Bam*HI. The resulting 2.0-kb DNA fragment was ligated into the *Bam*HI site of pHSG398sacB and the new plasmid named pHSG398sacBhrcC. To create a *hrcC*-mutation in pHSG398sacBhrcC, the EZ:TN™ Transposome™ system (Epicentre) was used according to the manufacturer’s instructions. Transposon inserted sites in the resulting plasmids were analyzed using nucleotide sequence analysis according to the manufacturer’s instructions and a plasmid, pHSG398sacBhrcCKm, into which the transposon was inserted into *hrcC* was selected.

### 3. Construction of Plasmids for Complementation of ΔaldH with *aldH* Originating from Pseudomonads

A 2.0-kb DNA fragment, designated Pc-aldH, containing the predicted promoter region and the open reading frame of *aldH* from SPC9018, was amplified by PCR from the genomic DNA of SPC9018 with the primers Kpn-Pc-aldH-Fw and Kpn-Pc-aldH-Rv. The *Kpn*I-digested Pc-aldH was ligated into the K*pn*I site of pUFR043 to create paldH. The pHSG398 was digested with *Sau*3AI and the resulting 1.1-kb fragment, including a chloramphenicol-resistance gene, was ligated into *Bam*HI-site of pUC118 resulting in pUC118-cml. A 1.1-kb fragment from *Sac*I-digested pUC118-cml was ligated into the *Sac*I-site of paldH, thereby creating pPc-aldH.

A 2.8-kb DNA fragment, designated Pv-aldH, containing the predicted promoter region and the open reading frame of *aldH* from Pv9504, was PCR-amplified from the genomic DNA of Pv9504 with the primers Bam-PV4-Fw and Bam-PV4-Rv. The *Bam*HI-digested Pv-aldH was ligated into the *Bam*HI site of pLAFR3, creating pPv-aldH.

A 2.0-kb DNA fragment, designated Pst-aldH, containing the predicted promoter region and the open reading frame of *aldH* from *P. syringae* pv. *tomato* strain DC3000, was PCR-amplified from the genomic DNA of *P*. *syringae* pv. *tomato* strain DC3000 with the primers Kpn-Pst-Fw and Kpn-Pst-Rv. The *Kpn*I-digested Pst-aldH was ligated into the K*pn*I site of pUFR043 to create pPst. A 1.1-kb fragment from *Sac*I-digested pUC118-cml was ligated into the *Sac*I-site of pPst, creating pPst-aldH.

A 2.4-kb DNA fragment, designated Pa-aldH, containing the predicted promoter region and the open reading frame of *aldH* from *P*. *aeruginosa* strain PAO1, was amplified by PCR from the genomic DNA of *P*. *aeruginosa* strain PAO1 with primers Pa-Fw and Pa-Rv. The *Xho*I-digested 2.0 kb Pa-aldH fragment was ligated into the *Xho*I site of pBbad22K resulting in pBbad22K-aldH. The plasmid pUC118-cml was digested with *Kpn*I and ligated into pBbad22K-aldH, creating pPa-aldH.

### 4. Construction of Plasmids for Complementation of Δpat with *pat* Originating from SPC9018 and Pv9504

A 1.5-kb DNA fragment, designated Pc-pat, containing the predicted promoter region and the open reading frame of *pat* from SPC9018, was amplified by PCR from the genomic DNA of SPC9018 with the primers delta5-1-Fw-Kpn and Bam-D4-2-Fw. The *Kpn*I- and *Bam*HI-digested Pc-pat was ligated into the K*pn*I and *Bam*HI sites of pUFR043 to create ppat. A 1.1-kb fragment from *Sac*I-digested pUC118-cml was ligated into the *Sac*I-site of ppat, thereby creating pPc-pat.

A 1.2-kb DNA fragment, designated Pv-pat, containing the predicted promoter region and the open reading frame of *pat* from Pv9504, was amplified from the genomic DNA of Pv9504 using PCR with the primers Kpn-PV-ORF5-RV and Bam-PV-ORF5-FW. The *Bam*HI- and *Kpn*I-digested Pv-pat was ligated into the *Bam*HI site of pUFR043 to create ppvpat. A 1.1-kb fragment from *Sac*I-digested pUC118-cml was ligated into the *Sac*I-site of ppvpat, thereby creating pPv-pat.

**Table S1:** List of bacteria analyzed and the accession numbers of the nucleotide sequences of *aldH* of bacteria deposited in the DDBJ/GenBank international nucleotide sequence database.

Bacteria	DDBJ Accession Number	Reference or Source
***P. cichorii***		
SPC9018	AB433910	[6]
MAFF730054	AB530808	This study
SPC9037	AB530809	This study
PCL2001	AB530810	This study
MAFF211387	AB530811	This study
MAFF301158	AB530812	This study
MAFF301374	AB530813	This study
MAFF302094	AB530814	This study
MAFF302698	AB530815	This study
KH5	AB530816	This study
MAFF302152	AB530817	This study
***P. viridiflava***		
Pv9504	AB530818	[6]

**Table S2:** Virulence of *P. cichorii* strains on the *Asteraceae* plants and the accession numbers of the nucleotide sequences of *ndfH* and *rbcL* deposited in the DDBJ/GenBank international nucleotide sequence database.

Plant Species	*P. cichorii* Strains					DDBJ Accession Number	
	SPC9018	SPC9018-hrcC	ΔaldH	Δpat		*ndhF*	*rbcL*
*Bidens laevis*	V	V	V	V		AB530917	AB530951
*Bellis perennis*	V	NV	NV	NV		AB530918	AB530952
*Calendula officinalis*	V	V	NV	NV		AB530919	AB530953
*Callistephus chinensis*	V	V	V	V		AB530920	AB530954
*Centaurea cyanus*	V	V	NV	V		AB530921	AB530955
*Centaurea macrocephala*	V	V	V	V		AB530922	AB530956
*Leucanthemum vulgare*	V	V	NV	V		AB530923	AB530957
*Leucanthemum maximum*	V	NV	NV	NV		AB530924	AB530958
*Coreopsis tinctoria*	V	NV	NV	V		AB530925	AB530959
*Cosmos bipinnatus*	V	V	V	V		AB530926	AB530960
*Crepis rubra*	V	NV	NV	NV		AB530927	AB530961
*Dahlia pinnata*	V	NV	NV	V		AB530928	AB530962
*Echinacea purpurea*	V	NV	NV	NV		AB530929	AB530963
*Echinops ritro*	V	V	NV	V		AB530930	AB530964
*Felicia heterophylla*	V	V	V	V		AB530931	AB530965
*Gaillardia aristata*	V	NV	NV	V		AB530932	AB530966
*Gazania splendens*	V	V	NV	V		AB530933	AB530967
*Helianthus annuus*	V	V	V	V		AB530934	AB530968
*Xerochrysum bracteatum*	V	V	NV	V		AB530935	AB530969
*Leucanthemum paludosum*	V	V	V	V		AB530937	AB530971
*Leontopodium alpinum*	V	V	V	V		AB530938	AB530972
*Rudbeckia hirta*	V	NV	NV	V		AB530939	AB530973
*Jacobaea maritima*	V	V	V	V		AB530940	AB530974
*Pericallis cruenta*	V	V	NV	V		AB530941	AB530975
*Tagetes patula*	V	V	V	V		AB530942	AB530976
*Zinnia violacea*	V	V	V	V		AB530943	AB530977
*Arctium lappa*	V	V	V	V		AB530944	AB530978
*Chrysanthemum coronarium*	V	V	V	V		AB530945	AB530979
*Cichorium intybus*	V	NV	NV	NV		AB530946	AB530980
*Cynara scolymus*	V	V	V	V		AB530947	AB530981
*Lactuca sativa*	V	V	V	V		AB530948	AB530982

V: the strain was virulent to the species; NV: the strain was not virulent to the species.

**Table S3:** Strains and plasmids used in this study.

	Relevant Characteristics	Ref. or Source
***E. coli***		
DH5α	*recA1 endA1 gyrA96 thi-1 hsdR17supE44*, *Δ(lac) U169 (**φ80lac**ΔM15*)	Takara
DH5α-aldH	Transformant of DH5α with pUC118-aldH, Ap^r^	This study
***P. cichorii***		
SPC9018	Wild-type	[5]
ΔaldH	*aldH*-deleted mutant of SPC9018, Km^r^	This study
ΔaldH (Pc-aldH)	Transformant of ΔaldH with pPc-aldH, Km^r^, Cm^r^	This study
ΔaldH (Pv-aldH)	Transformant of ΔaldH with pPv-aldH, Km^r^, Tc^r^	This study
ΔaldH (Pst-aldH)	Transformant of ΔaldH with pPst-aldH, Km^r^, Cm^r^	This study
ΔaldH (Pa-aldH)	Transformant of ΔaldH with pPa-aldH, Km^r^, Cm^r^	This study
Δpat	*pat*-deleted mutant of SPC9018, Km^r^	This study
Δpat (Pc-pat)	Transformant of Δpat with pPc-pat, Km^r^, Cm^r^	This study
Δpat (Pv-pat)	Transformant of Δpat with pPv-pat, Km^r^, Cm^r^	This study
SPC9018-hrcC	*hrcC*-deleted mutant of SPC9018, Km^r^	This study
SPC9018-L	*hrpL*-deleted mutant of SPC9018, Km^r^	[6]
***P. viridiflava***		
Pv9504	BS type	[6]
***P. syringae* pv. *tomato***		
DC3000		[11]
***P. aeruginosa***		
PAO1		[13]
Plasmid		
pUC118	Amp^r^	Takara
pHSG398	Cm^r^	Takara
pLAFR3	pLAFR1 containing *Hae*II fragment of pUC8	[36]
pUFR043	Cosmid derivative of pUFRO42, *IncW*, *Mob*’ *lacZα*, Gm^r^, Km^r^	[37]
pBbad22K	Derivative of pBAD22, *mob*, *rep*, *araC*, Km^r^	[38]
pUCK191	pUC18 derivative containing Km^r^ from Tn903	[33]
pUCD800	pUCD5 derivative containing *sacB*, Km^r^	[34]
phrpFoperon	A 3.6 kb PCR fragment containing the *hrpF* operon from SPC9018 genomic DNA	[6]
pUC118-cml	A 1.1 kb *Sau*3AI-digested pHSG398 in pUC118, Ap^r^, Cm^r^	This study
pPc-aldH	A 2.0 kb PCR-fragment containing *aldH* of SPC9018 and *Sac*I-digested 1.1 kb fragment of pUC118-cml in pUFR043	This study
pPv-aldH	A 2.8 kb PCR-fragment containing *aldH* of Pv9504 in pLAFR3	This study
pPst-aldH	A 2.0 kb PCR-fragment containing *aldH* of DC3000 and *Sac*I-digested 1.1 kbp fragment of pUC118-cml in pUFR043	This study
pPa-aldH	A 2.4 kb PCR-fragment containing *aldH* of PAO1 and *Kpn*I-digested 1.1 kb fragment of pUC118-cml in pBbad22K	This study
pPc-pat	A 1.5 kb PCR-fragment containing *pat* of SPC9018 and *Sac*I-digested 1.1 kb fragment of pUC118-cml in pUFR043	This study
pPv-pat	A 1.2 kb PCR-fragment containing *pat* of Pv9504 and *Sac*I-digested 1.1 kb fragment of pUC118-cml in pUFR043	This study

**Table S4:** List of primers used in this study.

Name	Sequence ^a^
Kpn-D4-1-Fw	5'-GGGGTACCACAGTTTTGTCCCTAAACCCG-3'
Bam-D4-1-Rv	5'-CGGGATCCGGCGTCCACAAAAAAGAGCG-3'
Bam-D4-2-Fw	5'-CGGGATCCTCACATCGGTATCTCCTGTTG-3'
Sal-D4-2-Rv	5'-GCGTCGACGCTATGATCATTCATCCTCAGC-3'
Bam-Km1	5'-CGGGATCCGGTACCCCCCCGCGCCTGATGC-3'
Bam-Km2	5'-CGGGATCCGGTACCCCCCCGCGCCTGATGC-3'
Sal-SacB1	5'-CGACGCGTCGACGGATCCTTTTTAACCCATC-3'
Sal-SacB2	5'-CGACGTCGACTGCAGTTCACTTACACCGC-3'
delta5-1-FW-Kpn	5'-GGGGTACCTGCCATCTGATGCTTTGAAAG-3'
delta5-1-RV-Bam	5'-CGGGATCCTCACATCGCTTCGAGATCGTCTTCAG-3'
delata5-2-Fw-Bam	5'-CGGGATCCTTCGCCAGCGTTGAAAAAAGGG-3'
delata5-2-Rv-Sal	5'-GCGTCGACGCGATTCGTTCCTGCCGCTATC-3'
Kpn-Pc-aldH-Fw	5'-GGGGTACCCCCGCATCAAACCGGTCATGG-3'
Kpn-Pc-aldH-Rv	5'-GGGGTACCGTCAGACGATAGGCTGGTC-3'
Bam-PV4-Fw	5'-CGGGATCCAAGCTATGATTAATCATCCAC-3'
Bam-PV4-Rv	5'-CGGGATCCTCAAGCGATCGGCTGATCACTC-3'
Kpn-Pst-Fw	5'-GGGGTACCCCCGCATCAAGCCGGTGATGG-3'
Kpn-Pst-Rv	5'-GGGGTACCTCACGCGACAGGCTGATC-3'
Pa-Fw	5'-GCTACGCGCCTGCTGCTACGGGC-3'
Pa-Rv	5'-GACCGCCTACGCCGCTGCCGCAG-3'
Kpn-PV-ORF5-RV	5'-GGGGTACCTCAGACAGCCTCCGATACGTG-3'
Bam-PV-ORF5-FW	5'-CGGGATCCGGTGGCATCACAACTGCGTATC-3'
SEMI-Back	5'-CTCACCGTTGACCAGACGC-3'
SEMI-Front	5'-GTCCAGCACTTGCTGGAGC-3'
ORF5-RT-Fw	5'-GGGGCCAACTCGCCGGTTAC-3'
aldH-Fw1	5'-GCGATTCGTTCCTGCCGCTATC-3'
aldH-Rv	5'-CCGCTCTTTTTTGTGGACGCCGG-3'
16S-rRNA-Rv	5'-AAATTCCACCACCCTCTGC-3'
16S-rRNA-FwndhF-11FW	5'-GCCTAGGTCGGATTAGCTAG-3'5'-GGGYTGGGACTTCTTCTTTTYCC-3'
ndhF-22RV	5'-CCSCCKACYSATTTAATAACC-3'
1-1	5'-ATGTCACCACAAACAGAGACTAAAGC-3'
NN3-2	5'-GCAGCAGCTAGTTCCGGGCTCCA-3'
ndhF-11RV	5'-TAGGYGAATACAACCAACTATC-3'
ndhF-22FW	5'-TTGCYTGTTTTTGGTCNAAAGATG-3'
1-2FW	5'-CAGTACTTCCATGTTGG-3'
1-2RV	5'-TATCCAACAAGAGTTTCC-3'

^a^ Restriction enzyme sites in the primer sequence are underlined: *Bam*HI GGATCC, *Eco*RI GAATTC, *Kpn*I GGTACC, and *Sal*I GTCGAC.
